# A dataset of 120 GHz millimeter-wave radar vital signals with synchronized reference recordings

**DOI:** 10.1038/s41597-026-07016-6

**Published:** 2026-03-13

**Authors:** Ruochen Wu, Laura Miro, Albert Aguasca, Antoni Broquetas, Cosme Garcia, Montse Najar

**Affiliations:** 1 CommSensLab-UPC, recognized as a consolidated research group by the Generalitat de Catalunya GRC-01415, Department of Signal Theory and Communications, Universitat Politècnica de Catalunya, 08034 Barcelona, Spain; 2 Signal Processing and Communications Group, recognized as a consolidated research group by the Generalitat de Catalunya 2021 SGR 01033, Department of Signal Theory and Communications, Universitat Politècnica de Catalunya, 08034 Barcelona, Spain; 3 INEDIT Research Group on Innovation Health Economics and Digital Transformation, Institut de Recerca Germans Trias i Pujol, 08916 Badalona, Spain; 4 Directorate of Healthcare Strategy and Innovation, Hospital Universitari Germans Trias i Pujol, 08916 Badalona, Spain; 5 Cardiology Department, Hospital Universitari Germans Trias i Pujol, 08916 Badalona, Spain

## Abstract

Millimeter-wave (mmWave) radar is widely recognized as a critical tool for contactless, continuous human sensing across multiple scenarios. Yet, there is a lack of high-quality datasets with synchronized reference measurements, especially at higher frequencies, which are essential for advancing signal processing methods and improving the retrieval of vital parameters. To address this gap, we introduce a mmWave radar vital signal dataset collected with synchronized reference recordings. The dataset is derived from measurements acquired using a custom-built, noncommercial radar system developed by CommSensLab-UPC specifically for biomedical applications. The implemented Frequency-Modulated Continuous Wave (FMCW) radar system operates at 120 GHz within the industrial, scientific, and medical (ISM) band. In parallel, a monitoring system records reference physiological signals, including electrocardiograms, respiratory traces, pulse waveforms, and blood pressure values. Under a predefined protocol, a professional clinician collected data from 24 healthy subjects under two scenarios. The release of this dataset aims to facilitate the development and validation of advanced radar signal processing algorithms, thereby enhancing the contribution of radar technologies to hemodynamic monitoring and autonomic nervous system assessment.

## Background & Summary

Radar technology has found widespread application in the biomedical field, with radar sensors increasingly gaining attention in human sensing, particularly for vital sign monitoring, due to their compact size, low cost, and contactless nature^1–3^. In cardiopulmonary activity monitoring, traditional methods such as electrocardiography (ECG) require physical electrode connections to the patient to capture electrical signals generated by atrial and ventricular activity during each heartbeat^4^. However, in scenarios involving newborns^5^, burn patients^6,7^, isolated individuals^8^, or in smart home and in-cabin environments^9^, remote monitoring presents a more suitable solution. Recent studies have demonstrated the robustness of millimeter-wave radar in monitoring cardiopulmonary activity and have successfully extended its application to multi-subject monitoring^10,11^.

Initial evaluations of feature extraction algorithms are typically conducted using small, custom datasets that ensure controlled experimental activity. However, incorporating diverse datasets into the validation process significantly strengthens the methodology by enabling performance assessments across various scenarios and revealing potential limitations. Despite a recent increase in published studies, public datasets with reference measurements remain limited^12^. Several existing datasets offer short and controlled recordings with minimal noise, acquired using low/mid frequency Continuous Wave (CW) and Frequency-Modulated Continuous Wave (FMCW) radars operating at 10 GHz, 24 GHz, and 70 GHz^13–15^, making them well-suited for early-stage testing of algorithm performance. It is also noteworthy that a 24 GHz six-port-based CW radar vital signals dataset with long recording durations have been available^16^. In addition, some studies have introduced radar human datasets covering different recording scenarios, contributing to multi-target monitoring and search and rescue missions^17,18^. However, most of the datasets are derived from low- to mid-frequency commercial radar systems, which were originally designed for security and automotive applications rather than biomedical use^19–22^. Moreover, the available datasets are notably heterogeneous in terms of the radar systems used, and they provide signals captured under varying scenarios, positions, acquisition durations, reference frameworks, and other parameters.

Given the current scarcity of publicly available datasets related to radar-based vital signs in the field of contactless human sensing, we present a dataset of vital signals from healthy human subjects acquired using a custom-designed and optimized millimeter-wave (mmWave) FMCW radar system-on-chip (RSoC) developed for biomedical applications. The radar operates at a higher frequency of 120 GHz compared to previous datasets. The proposed dataset comprises radar vital signal data from 24 subjects, each one in two different scenarios, spanning a total duration of 5760 seconds. Simultaneously, we acquired reference sensor signals, including electrocardiogram (ECG) signals, photoplethysmography (PPG) signals, respiratory sensor signals, and sphygmomanometer readings, to serve as benchmark references for the corresponding components in radar vital signals. By releasing this dataset, we aim to provide detailed information to researchers to be able to understand and properly exploit the dataset to support the research of new processing techniques and innovative applications. For example, the presented dataset can serve as a testing ground for advanced signal processing and intelligent algorithms aimed at removing motion artifacts and extracting vital signs from body movements. Moreover, future work may involve extending the dataset to include clinical scenarios and integrating radar data with other contactless modalities to enhance the robustness of healthcare monitoring systems.

## Methods

### Participants

Participants were recruited based on strict inclusion and exclusion criteria. Eligible individuals were healthy adults of legal age, selected to ensure a sample that was diverse in terms of age, height, and weight, and that achieved a balanced distribution of sexes. Individuals with any diagnosed cardiorespiratory dysfunction or those using pacemakers were excluded. To avoid any potential conflict of interest, people working or engaging in academic activities in the laboratory responsible for the study, as well as students, family members of the participating researchers, or any individual with an economic, hierarchical, functional, or organizational relationship with the researchers, were not eligible. Participants were contacted face-to-face or via email. Prior to participation, each subject was informed about the study’s objectives, the procedures to be performed, and the potential risks involved.

A total of 24 healthy participants were included in the study, with a mean age of 38.33  ± 14.64 years. The mean body weight, height, and body mass index (BMI) were 67.96  ± 12.86 kg, 1.71  ± 0.1 m, and 23.02  ± 3.04 kg/m^2^, respectively. Thirteen participants were male. Table 1 summarizes the demographic and anthropometric characteristics of the study.

**Table 1:** Subjects’ Information.

Subject ID	Age	Sex^*a*^	Weight (kg)	Height (m)	BMI^*b*^ (kg/m^2^)
VS01	28	M	60	1.69	21.01
VS02	27	M	92	1.85	26.88
VS03	30	M	70	1.68	24.80
VS04	30	M	82	1.79	25.59
VS05	25	M	70	1.78	22.09
VS06	30	M	75	1.91	20.56
VS07	27	F	55	1.62	20.96
VS08	62	M	83	1.70	28.72
VS09	48	M	78	1.80	24.07
VS10	53	F	55	1.59	21.76
VS11	59	M	82	1.85	23.96
VS12	28	F	65	1.75	21.22
VS13	61	F	53	1.59	20.96
VS14	44	F	77	1.69	26.96
VS15	58	F	63	1.67	22.59
VS16	27	F	65	1.64	24.17
VS17	25	F	46	1.60	17.97
VS18	25	M	75	1.78	23.67
VS19	43	M	57	1.73	19.05
VS20	59	M	80	1.84	23.63
VS21	25	M	74	1.73	24.73
VS22	24	F	52	1.68	18.42
VS23	57	F	77	1.63	28.98
VS24	25	F	45	1.51	19.74

^*a*^ M: male, F: female. ^*b*^ Body mass index.

### Ethics statement

The study was approved by the ethics committee of the Universitat Politècnica de Catalunya  ⋅ BarcelonaTech (Identification code: 2024-028). Informed consent for participation and data sharing was obtained and signed in accordance with the ethical principles governing the study. All data have been fully anonymized, ensuring that no information can be traced back to individual participants. Moreover, subjects provided relevant biologic information, including age, sex, weight, and height for further analysis and each subject was assigned a unique identifier.

### Radar system

**Fig. 1:**
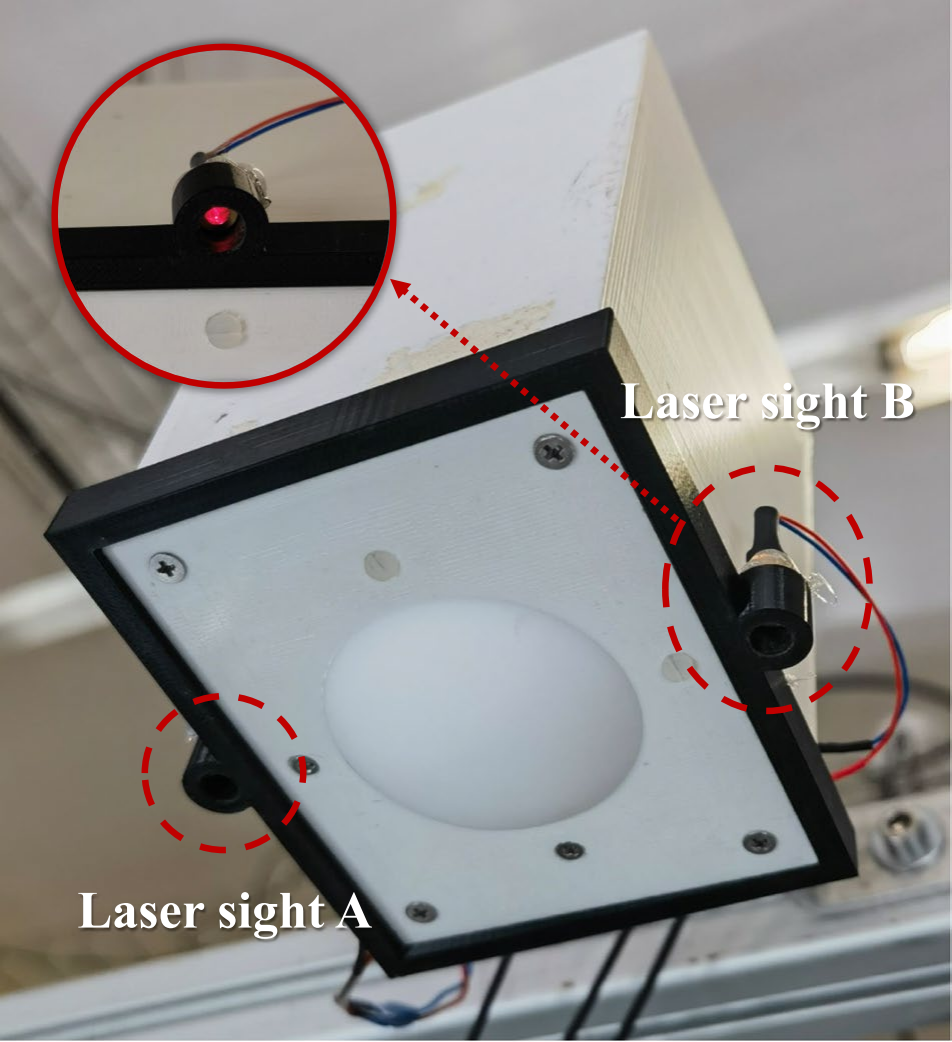
Laser sights integrated on both sides of the exterior of the radar head.

**Fig. 2:**
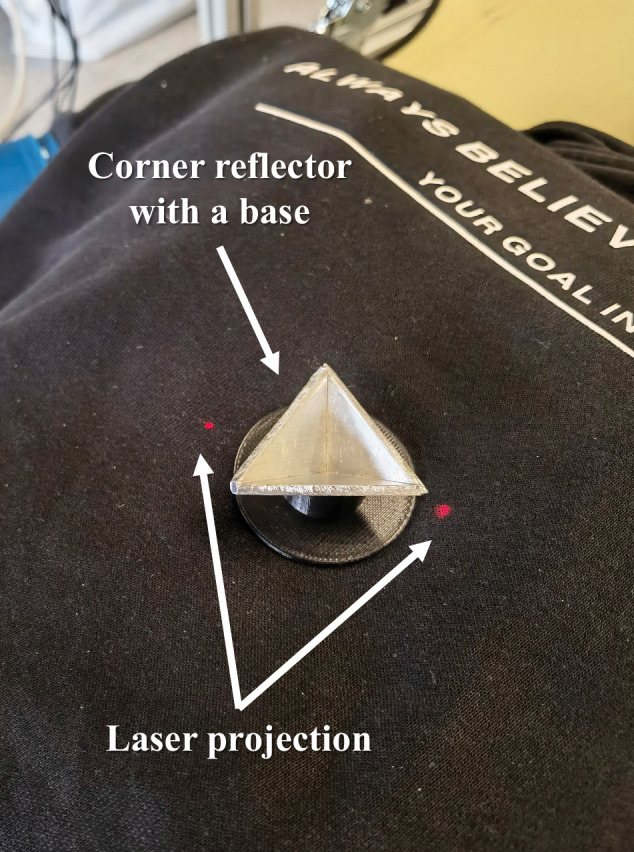
Laser projection with a corner reflector for radar pointing before data acquisition.

The mmWave RSoC used in this study was specifically designed and developed in our laboratory (CommSensLab-UPC) for vital sensing applications^23^. A 120 GHz transceiver chip manufactured by the company Indie (indie.inc) has been integrated, enabling high-range resolution by operating on the 122–123 GHz industrial, scientific, and medical (ISM) band. At this very high frequency, clothing and other textile materials offer an excellent transmissivity and the short wavelength *λ* = 2.45 mm allows to achieve an excellent phase based micro-metric body motion sensing^24^. The radar transmission frequency is governed by a phase-locked loop (PLL) with a direct digital synthesizer (DDS) serving as the reference. The DDS is programmed to generate a FMCW transmit waveform that linearly sweeps the frequency range from 122 to 123 GHz. The radar receiver mixes the received echo with a sample of the transmitted signal, generating a beating tone signal whose frequency and phase are proportional to the distance of the reflecting object, which is then digitized for subsequent preprocessing to extract the phase evolution. In addition, to enhance antenna beam alignment, the system is equipped with laser pointers on both sides of the antenna, providing a visual aid for beam positioning. Figure 1 shows the integration of the lasers equipped on the radar housing, whereas Fig. 2 illustrates the laser projection pattern on the body.

The main parameter settings of the radar are listed in Table 2^25^. In practical measurements, a lens positioned in front of the radar generates a narrow beam that defines a precise observation point on the human body, with a diameter of 3.5 centimeters at a distance of 1 meter. The lens enhances the antenna gain substantially, allowing for the detection of echoes with a high signal-to-noise ratio (SNR) even at distances of several meters. The combination of the directional antenna and high bandwidth provides exceptional range resolution, effectively suppressing nearby unwanted movements that could interfere with vital signal detection. The measurement duration is 2 minutes according to the acquisition protocol.

**Table 2:** Main Parameter Settings of the Radar.

Parameter	Value
Center Frequency (*f*_0_)	122.5 GHz
Radar Nominal Bandwidth^1^ (*B*)	1 GHz
Antenna Beamwidth (*θ*_3dB_)	2^°^
Radar Range Resolution (Δ*r*)	$\frac{c}{2B}=$ 150 mm
Wavelength (*λ*)	$\frac{c}{{f}_{0}}=$ 2.449 mm
Chirp Repetition Period (*T*_frame_)	3 ms
Chirp Slope Time (*T*)	1.5 ms

1: Specified by the industrial, scientific, and medical (ISM) band.

Additionally, it should be noted that although the RSoC is primarily designed for the 122–123 GHz ISM band, it supports bandwidth extension up to 7 GHz. In our laboratory experiments, the risk of interfering with other equipment was negligible due to the radar’s low transmission power and narrow beamwidth. Thus, we used a 3 GHz bandwidth, which yielded a finer range resolution of 5 centimeters, thereby effectively distinguishing the target from unwanted environmental reflections.

To achieve precise original vital signal acquisition, the beat tone is first digitized, followed by windowing the signal to smooth initial and final transients and reduce spectral sidelobes. Subsequently, the frequency-domain range cells of interest are identified, and the complex values of each radar frequency sweep are extracted. Phase extraction and unwrapping are then performed to accurately capture the micro-motion of the subject, further the phase is converted into distance using the wavelength *λ*:

1 $$R\left(t\right)=\frac{\phi \left(t\right)\lambda }{4\pi }.$$

This approach enables the differentiation of micron-scale displacements that vary over time, including chest vibrations caused by respiration and heartbeats. The details of the radar system, signal processing pipeline, and quantitative experimental evaluations can be found in^25^. Specifically, the measurement error in radar sensors is primarily attributed to phase noise present in the target echo signal. When instead of a human subject the radar observes a static object in the same conditions of range and echo power the obtained phase is almost constant, with a residual phase noise expressed as standard deviation of 0.0024 rad^24^. This value corresponds to a radar instrumental range precision expressed as standard deviation of *σ* = 0.47 *μ*m.

In conclusion, the mmWave radar measured data were recorded by means of a high speed digitizer. The digitizer is managed by a C++ based executable. The acquired signals were processed by MathWorks MATLAB R2024b.

### Reference-standard measurements

The reference patient monitoring system used in this study is a Mindray ePM10 multi-parameter vital signs monitor. The system supports the integration of optional modules to measure specific parameters, enabling comprehensive and synchronized monitoring of physiological signals. The complete system comprises the ePM10 monitor, an ECG device, a PPG, a respiration sensor and a sphygmomanometer. To ensure accurate temporal alignment across all signals, the ePM10 device was synchronized using the same Network Time Protocol (NTP) as the radar. This common synchronization framework is critical for correctly aligning the acquisition of multi-modal data and ensuring reliable subsequent analyses.

The device was provided by the Germans Trias i Pujol Hospital and it has its measurements validated by the Hospital’s Electromedical Unit, in addition to holding the European CE mark, certifying its safety.

#### ECG

The ePM10 features a three-channel electrocardiogram for recording the heart’s electrical activity. Electrodes are attached according to clinical standards, using four color-coded leads based on Einthoven’s triangle. Snap electrodes are used for the recordings, with a new set provided for each subject to ensure proper hygiene. Leads II and III are captured, allowing lead I to be derived by subtracting lead III from lead II. Moreover, lead V1 is also recorded, offering critical insights into the electrical activity of the right ventricle and anterior cardiac regions, which can further enhance the overall cardiac assessment. The raw ECG data are digitized at a sampling rate of 500 Hz. During post-processing, a high-pass filter with a cutoff frequency of 0.5 Hz is applied to the ECG signals to remove baseline wander and low-frequency noise.

#### Respiratory sensor

The system includes an impedance respiratory sensor designed to capture breathing activity. This sensor, integrated within the electrode configuration, records respiratory rate and amplitude. The respiratory signal is sampled at 256 Hz. The respiratory waveform is unitless, representing relative changes in thoracic impedance during breathing. Its significance lies in its pattern in relation to the respiratory cycle rather than in any absolute numerical measurement.

#### PPG

A PPG sensor is integrated into the ePM10 to enable noninvasive monitoring of blood oxygen saturation ($S{p}_{{{\rm{O}}}_{2}}$) and pulse rate. The PPG sensor is placed on the left fingertip and captures the pulse wave signal. The sampling rate is 60 Hz.

#### Sphygmomanometer

The system has a traditional cuff-based sphygmomanometer module, placed on the right upper arm, that measures systolic (BPS), diastolic (BPD) and mean blood pressure (BPM) on demand. The last one can also be calculated using the following expression:

2 $${\rm{BPM}}={\rm{BPD}}+\frac{({\rm{BPS}}-{\rm{BPD}})}{3}.$$

### Acquisition protocol

**Fig. 3:**
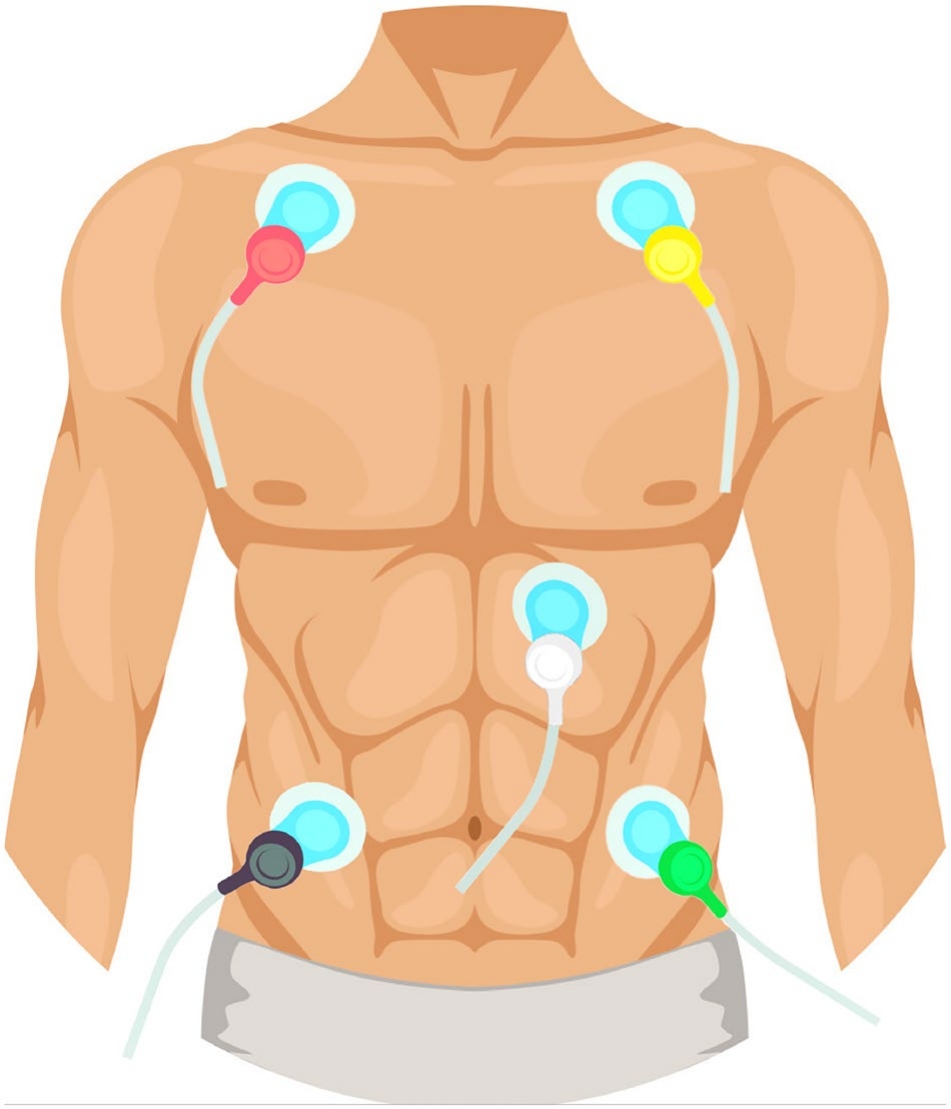
Schematic diagram of standard placement of ECG electrodes.

The entire process of signal and physiological data collection was carried out in the Antenna and Radar Research Laboratory of the Department of Signal Theory and Communications of the Universitat Politècnica of Catalunya (UPC). During the measurement process, two researchers oversaw the entire procedure, with one responsible for managing the radar system and the other for carrying out experimental protocol and managing the reference device. Prior to initiating the measurement, standard placement of the ECG electrodes was performed according to clinical guidelines as shown in Fig. 3, a cuff for blood pressure measurement was properly adjusted on the right arm of the subject to ensure accurate readings, and the pulse oximeter was placed on the index finger of the left hand.

**Fig. 4:**
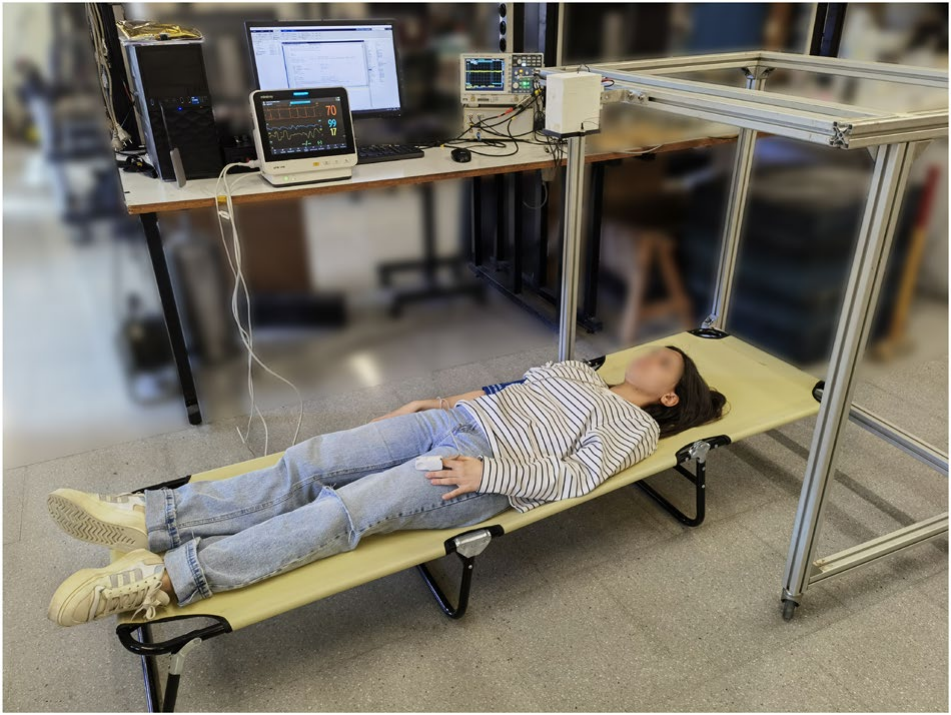
Laboratory measurement environment and its configuration. The subject depicted consents to the open publication of the image.

The subject was then positioned on a stretcher, lying supine with the upper body oriented toward the radar, as illustrated in Fig. 4. A corner reflector was placed over the xiphoid process, which was the region of interest (ROI), to correctly align the radar, and an initial 10-second capture was performed to verify that the signal was being acquired properly. The distance between the antenna and the ROI was around 0.6 meters during all measurements, and it was consistent across subjects due to the structure that secures the radar’s antenna. To enhance signal quality, the radar was oriented perpendicularly to the chest surface.

Following the preparation, the subject was allowed to rest for 1.5 minutes. This resting period allowed for autonomic recovery, i.e., the return of the sympathetic nervous system to its basal state, thereby minimizing the transient effects of adrenaline on the vital signs. This protocol ensures that the measured parameters reflect the subject’s true resting physiological conditions^26^. After the above procedures, the collection officially begins. Data were acquired under two conditions: a resting state and an apnea state.

#### Resting

The recording began under resting conditions and continued for 2 minutes to capture baseline physiological signals. Once the recording was complete, the subject’s blood pressure was measured.

#### Apnea

Following the resting state, the apnea acquisition was conducted. Initially, the subject was allowed to rest for 1.5 minutes. Data acquisition was initiated and maintained for 40 seconds under resting conditions. After this period, the subject was instructed to inhale and hold their breath for 10–20 seconds, then exhale and maintain normal breathing for 15–20 seconds, followed by another 10-second apnea period. After the apnea sequence, the subject remained at rest until the end of the 2-min acquisition, at which point blood pressure was measured once more.

Finally, all electrodes and the blood pressure cuff were removed, thereby concluding the entire procedure. It should be noted that the conditions were acquired in a fixed sequence, with resting always recorded before apnea. Although recovery periods were included to minimize transient effects, this fixed ordering remains a limitation and an order or carryover effect cannot be excluded. In addition, recordings were collected in a single session per participant, so test-retest reliability and time-of-day effects were not assessed.

**Fig. 5:**
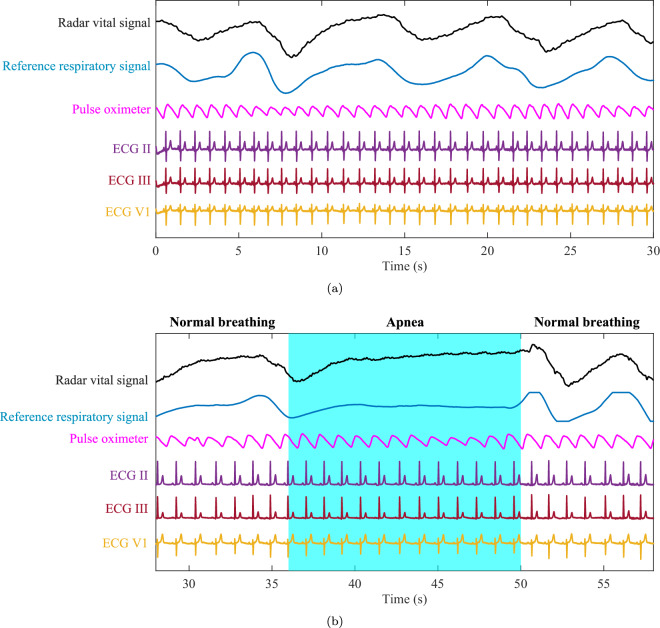
Exemplary signals of radar and ECG in different scenarios. (**a**) Resting. (**b**) Apnea.

Figure 5 presents representative excerpts of measured signals obtained from two different subjects under varying physiological states. Fig. 5a displays the raw signals recorded during a resting-state measurement, where the subject was engaged in normal breathing. The radar and reference signals clearly indicate a stable and calm breathing pattern. Similarly, Fig. 5b shows a segment of raw signals captured from a subject experiencing apnea. In this scenario, the subject transitions from normal breathing to a breath-holding phase and subsequently resumes normal respiration. The recorded signals reveal that the apnea episode lasted approximately 14 seconds. Moreover, in the apnea phase of the radar vital signal, weak heartbeat pulse motions can be observed, corresponding to the separated cardiac signal. Ground-truth measurements can be seen for both cases.

## Data Records

The dataset is publicly open access available online^27^ (10.21227/wq68-sv85). The dataset is organized such that each subject has a dedicated folder named after their unique subject ID. All data were saved to “.mat” file format. Additionally, a supplementary file named “Subject Information.xlsx” is also provided with the data, offering a comprehensive overview of the dataset structure and subject information. Within each of these folders, four separate files are provided, each corresponding to a different recording condition and measurement device.

For the radar data, two files labeled with the subject ID followed by “Resting” and “Apnea” contain the displacement data extracted from the mmWave radar (*VitalSig*) for the respective conditions in millimeters. Moreover, each radar data file includes a variable that represents the sampling frequency of the radar signal (*Radar.fs*) and the structure of its corresponding time series (*Radar.t_frame*). There is also a cell array named *MeasurementInfo*, which encapsulates three key elements: the subject ID, the timestamp of the recording, and the specific state scenario.

For the reference data, two further files are provided with the suffix “_Mindray” appended to the subject ID (i.e., “VS01_Resting_Mindray” and “VS01_Apnea_Mindray"). These files comprise data obtained from the reference monitoring device. The reference device files include different variables as listed in Table 3.

**Table 3:** Overview of parameters from the reference system.

Variable	Full name
BPS	Systolic Blood Pressure
BPM	Mean Blood Pressure
BPD	Diastolic Blood Pressure
HR	Heart Rate
RR	Respiratory Rate
ecg_lead2	ECG Lead II
ecg_lead3	ECG Lead III
ecg_leadv1	ECG Lead V1
respiration	Respiratory Signal
pleth	Photoplethysmography Signal
Fs_ecg	ECG Sampling Frequency
Fs_resp	Respiration Sampling Frequency
Fs_pleth	PPG Sampling Frequency

## Technical Validation

### Synchronization validation

**Fig. 6:**
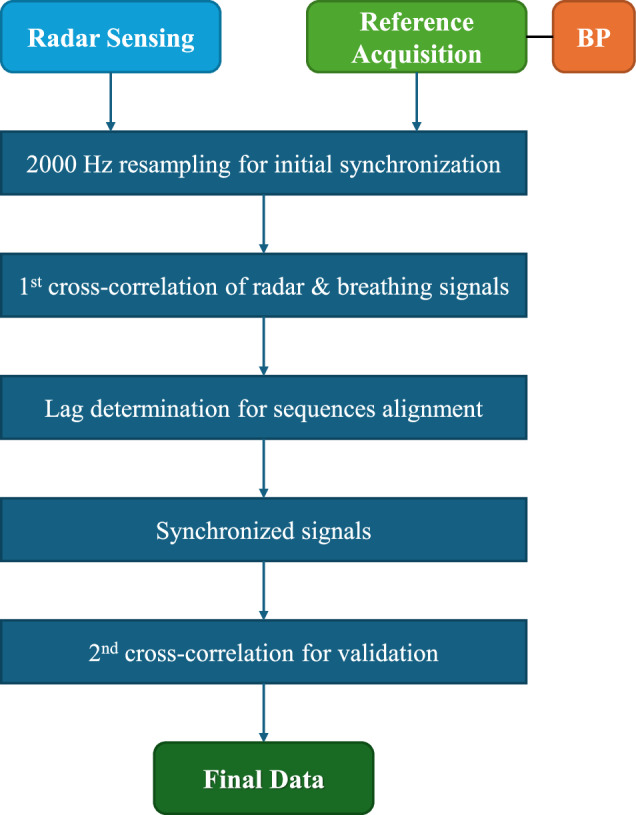
Block diagram of the data acquisition and signal synchronization process.

Prior to each experimental procedure, the reference time was synchronized using the NTP. However, a residual time deviation of up to 3 seconds relative to the system clock was still observed. To address this issue, we subsequently applied a alignment method, using the radar signal acquisition timestamps as an initial reference, to ensure accurate temporal synchronization. The entire data processing to obtain the synchronized data of the dataset is shown in Fig. 6.

**Fig. 7:**
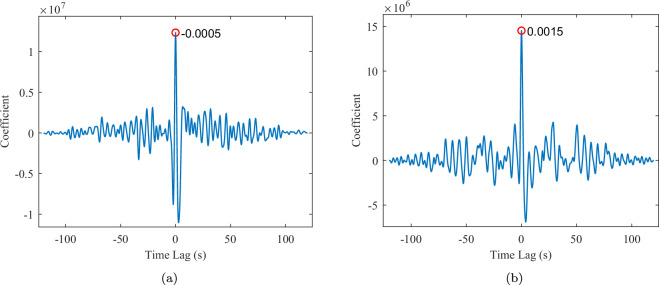
Cross-correlation of the synchronization under different scenarios for VS19. The maximum peak corresponds to the lag between radar and reference signals. (**a**) Resting. (**b**) Apnea.

After temporal alignment, cross-correlation^28^ is used between the radar vital signal and the reference respiratory signal to validate their synchronization, as the respiratory signal serves as the primary reference whose temporal dynamics closely correspond to those observed in the radar signal. Given the discrepancy in sampling frequencies between the radar and reference signals, both signals are resampled to a common frequency of 0.5 milliseconds, that is, 2000 Hz, prior to performing the correlation analysis. Figure 7 illustrates a exemplary cross-correlation plot for the same subject under resting and apnea conditions, respectively.

As shown in Fig. 7a, the maximum peak observed after alignment in the resting state is −0.0005 seconds, indicating that the radar signal exhibits a temporal offset of approximately 0.5 milliseconds relative to the reference signal. Whereas, under the apnea condition shown in Fig. 7b, the maximum peak of the cross-correlation is 1.5 milliseconds. This case reflects satisfactory synchronization between radar and reference signals.

**Fig. 8:**
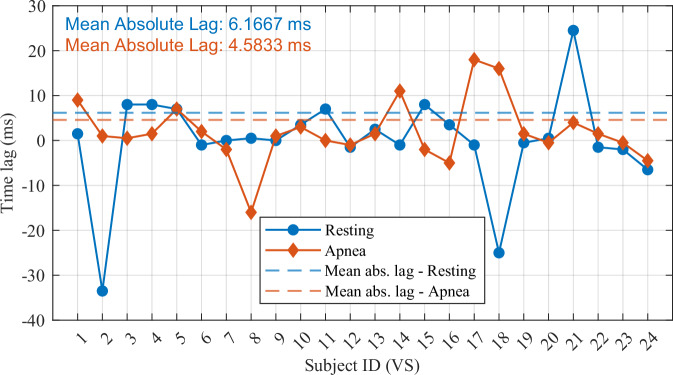
Synchronization time lag for each recording.

The cross-correlation result for all synchronized signals in the proposed dataset is shown in Fig. 8. In conclusion, the average absolute time offset for resting and apnea scenarios are 6.1667 milliseconds and 4.5833 milliseconds, respectively. When considering all scenarios combined, the overall average absolute time offset is 5.375 milliseconds. These results demonstrate a high degree of synchronization between the radar and reference signals following the time alignment process.

### Radar micro-motion sensing validation

To validate the surface micro-motion sensing capability of the radar without distortion or saturation, as required for cardiorespiratory monitoring, we conducted a experiment using a loudspeaker diaphragm driven by a triangle-wave signal. In this case, a function generator generates triangular and rectangular excitation waveforms at frequencies of 1.05 Hz and 1.28 Hz, respectively, which are used to drive the loudspeaker. The radar observed the diaphragm at a distance of 1.2 meters for a measurement duration of 9 seconds.

**Fig. 9:**
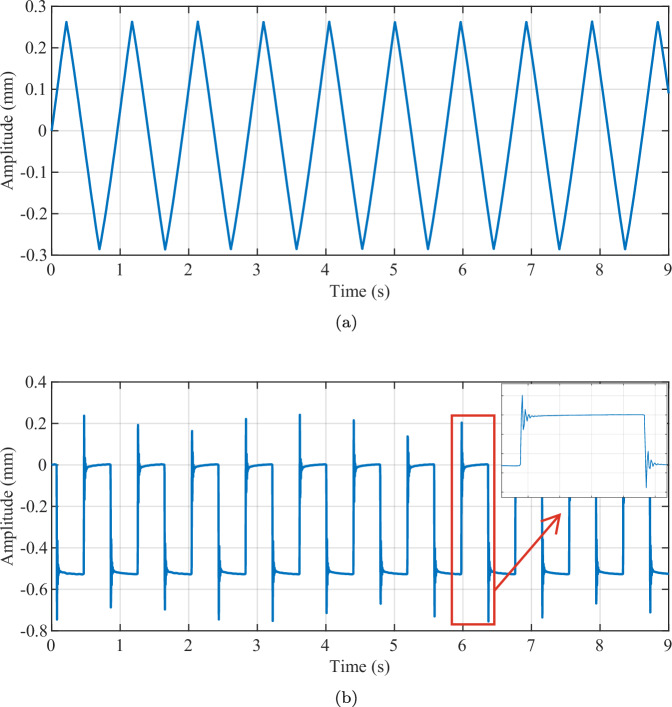
Radar observed diaphragm displacement signal of the loudspeaker. (**a**) 1.05 Hz triangle wave. (**b**) 1.28 Hz rectangular wave.

In the loudspeaker experiment illustrated in Fig. 9a, the radar micro-displacement signal exhibits a distinct triangular periodic waveform. The linear ramp characteristics and submillimeter-level peak-to-peak motion are accurately preserved, with no evidence of drift or clipping. As shown in Fig. 9b, the radar successfully captured the mechanical overshoot and ringing effects of the loudspeaker membrane at the rising and falling edges. These high-frequency features result from the physical inertia of the loudspeaker’s mass-spring system when driven by an abrupt step signal. The ability to resolve these rapid transient motions demonstrates that the radar possesses the required temporal resolution and high-frequency fidelity to detect complex morphological details in human vital signs.

The experimental result demonstrate that the radar accurately tracks sub-millimeter motion with minimal bias and distortion. Given that thoracic excursions are on the order of millimeters and cardiac micro-motion lies in the submillimeter regime, the observed fidelity indicates adequate dynamic range and accuracy for in-vivo cardiorespiratory waveform capture.

## Data Availability

Radar vital data were deposited into the IEEE DataPort database and are available at the following 10.21227/wq68-sv85.

## Code availability

The code used for the technical validation is available at https://github.com/Rc-W024/VS_DATASET. In addition, a reference script for radar breathing and cardiac signals separation is also included in the repository which can be used by configuring the subject and/or scenarios which shall be viewed. The code was written and tested using MATLAB R2024b for Microsoft Windows.
